# Boy Scouts and a Hospital Toy Service

**Published:** 1914-05-09

**Authors:** 


### Boy Scouts and a Hospital Toy Service.

It speaks well for the interest shown in local institutional life in Surbiton that at a recent "Toy Service," held at St. Matthew's Church, a number of toys, etc., was received so much in excess of what was expected that the Boy Scouts had to be called in with their trek carts in order to convey the toys to the local hospitals.

